# Driving Global Health equity and precision medicine through African genomic data

**DOI:** 10.1093/hmg/ddaf025

**Published:** 2025-04-30

**Authors:** Oyesola O. Ojewunmi, Segun Fatumo

**Affiliations:** 1 Precision Healthcare University Research Institute, Queen Mary University of London, Empire House, 67-75 New Road, London E1 1HH, United Kingdom; 2 MRC/UVRI and LSHTM Uganda Research Unit, Plot 51-59 Nakiwogo Road, PO Box 49, Entebbe, Uganda

**Keywords:** genomic diversity, precision medicine, African genomics, global health, polygenic risk score

## Abstract

Significant gaps persist despite the progress in raising awareness of genomic diversity and including individuals of African ancestry in genomic research. African populations remain underrepresented in genomic studies despite their deep evolutionary history, demographic diversity, and unique genetic architecture for gene discovery. This underrepresentation constrains the portability of findings from other populations to African settings due to the poor predictive performance of genetic scores. Consequently, it hinders global efforts in translational research, slows the progression of genomic medicine, and worsens health disparities—a missed opportunity for precision medicine globally. However, genuine prioritisation and expansion of genomic data collection from individuals of African ancestry can drive more equitable health solutions that benefit all populations. In this review, we highlight the opportunities presented by African genomic diversity, the urgent need for larger datasets and biobanks with diverse phenotypes from African populations, and recent developments in African genomic research.

## Introduction

Understanding the complex interplay between genetics and disease in diverse populations has become increasingly crucial in population genomics. This study area has provided valuable insights into human genetic diversity and potential paths for tailored healthcare. Africa, with its deep evolutionary history and rich genetic diversity, reflects its status as the origin of modern humans and represents an important frontier for unravelling novel genetic discoveries with great potential for advancing our knowledge of human biology and susceptibility to disease, offering an optimistic outlook for the future of genomic studies and precision medicine [1–3]. Whole genome sequencing of 426 individuals representing 50 ethnolinguistic groups across 13 African countries, resulted in the discovery of over 3 million previously unknown genetic variants [4]. However, there remains severe underrepresentation of African genomics in the global genomic data which has had far-reaching consequences, limiting gene discovery, understanding of disease biology, and the global efforts towards translational research. This represents a missed opportunity for precision medicine, not only for the African populations but also the global populations.

Large-scale population-based biobanks have been a rich resource for advancing genomic medicine and enhancing public health. However, most of the genome-wide association studies (GWASs) have primarily involved individuals of European ancestry (largely from Iceland, the UK, and the USA), who account for 86% of participants in GWAS Catalog. [1, 5–7]. Also, genomic biobanks, such as the UK biobank and the deCODE database, comprise mainly individuals of European ancestry. This lack of genetic diversity poses a significant limitation to the progression of personalised genomic medicine and worsens health disparities. Findings from European populations may not be replicable or transferable to others [8, 9] due to poor predictive accuracy of genetic models/scores in populations with diverse ancestries.

Despite increasing awareness about genomic diversity and inclusion, and notable progress made through the inclusion of individuals of African ancestry in many genomic research initiatives [10–15], generation of large-scale datasets from the African populations continues to lag and significant gaps persist. Even with the Eurocentric bias in the published GWAS, African-ancestry populations account for approximately 7% of all genotype–phenotype associations, indicating that inclusion of individuals of African ancestry can enhance identification of novel disease-causing variants [16]. How can we claim progress in global health when Africa—a continent with a median age of 19 and over 1.5 billion people [17]—remains underrepresented? Prioritising and expanding data collection from individuals of African ancestry is ethically, morally and economically imperative and can foster more equitable health solutions that benefit everyone. This review highlights the opportunities presented by African genomic diversity, the urgent need for larger datasets and biobanks with a wide range of phenotypes from African populations, and recent developments in African genomic research.

## Leveraging African genomic diversity for fine-mapping

GWASs have been pivotal in identifying SNPs associated with many diseases and traits. However, genome-wide association findings do not establish causation. One approach to explore whether association signals might be causal is fine-mapping—a statistical method that refines GWAS association signals to pinpoint variants most likely to have a direct impact on a trait. This method often integrates regulatory, protein-coding, and epigenomic annotations to enhance the identification of putative causal variants and their functional roles, though experimental validation is necessary to confirm causation.

**Figure 1:**
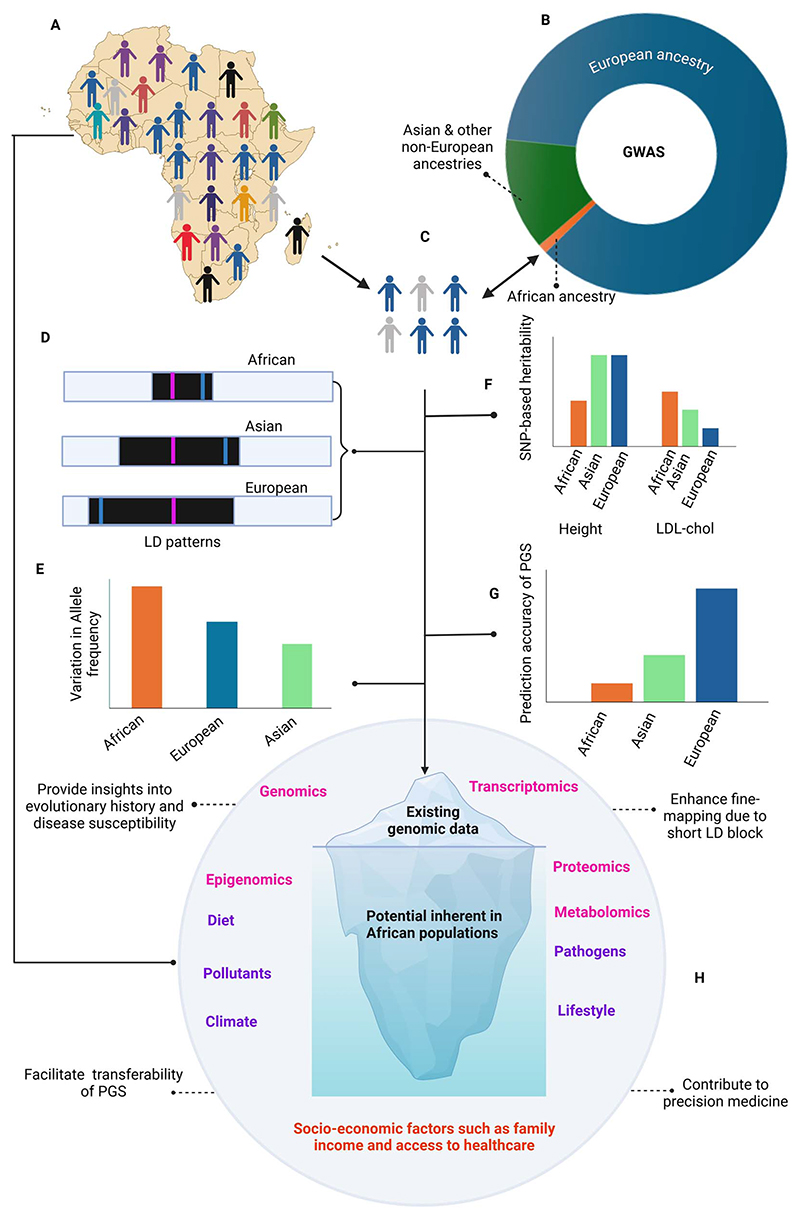
Genetic Diversity, Demographic Complexity, and the Underrepresentation of African Populations in Genomics. (A) Diverse African populations: This highlights the diverse African populations with deep evolutionary and demographic histories. (B) Ancestries in published GWASs: African populations are severely underrepresented (1.1%) compared to European ancestry (86%) and other non-European ancestries (12.9%), which include Asians, Hispanics/Latinos, and others [7, 8]. (C) African populations in current published GWAS: The representation of individuals of African ancestry in published GWAS is minimal. These individuals, predominantly African Americans, do not reflect the full diversity of African populations. (D) LD patterns across different ancestries*: The size of LD blocks varies by population. African populations have smaller haplotype blocks due to higher recombination rates, enabling finer resolution for pinpointing causal variants. (E) Variation in allele frequency*: African populations, with larger effective population sizes, harbour alleles that are rare or absent in other populations. As a result, their genomes contain significantly more single nucleotide variants (SNVs) than non-African genomes. (F) SNP-based heritability*: The heritability of complex traits and diseases in Africans differs significantly from other populations. For example, SNP-based heritability for height is lower in African populations, while LDL-cholesterol (LDL-chol) heritability is higher [22–24]. These differences reflect the combined impact of genetic architecture and environmental factors on heritability and polygenic scores. (G) Prediction accuracy of polygenic scores (PGS)*: Polygenic scores derived from European datasets perform poorly for many traits in African individuals. This lack of transferability limits the clinical applicability of PGS for African populations. (H) Factors underpinning African diversity: African diversity is shaped by a complex interplay of genomics, epigenomics, transcriptomics, proteomics, metabolomics and environmental factors such as diet, lifestyle, pathogens, climate, and pollutants. Current findings on African populations barely scratch the surface—the tip of the iceberg—representing only a fraction of the insights possible. Digging deeper is essential to generating datasets that are representative of African populations. Such efforts can uncover evolutionary histories, shed light on disease susceptibility and protection, enhance fine-mapping resolutions, improve the portability of PRS, and advance contributions to precision medicine. Additionally, social determinants such as family income and access to healthcare, play significant roles in shaping health outcomes in Africa. These factors should be integrated with genomic and other -omic data to better understand health and disease in African populations [14, 25]. *These figures were modified from references [8, 22–24, 26, 27].

The ability to identify causal variants from the GWAS hits depends on patterns of linkage disequilibrium (LD), which vary across populations. While the causal variant for a phenotype may be shared across populations, it can be tagged by different ancestry-specific variants. African ancestry populations, characterised by greater genetic diversity and shorter LD blocks compared to non-African populations, provide higher resolution for localising putative causal variants [18–21] (Fig. 1).

The out-of-Africa migration reduced genetic diversity and increased LD in non-African populations [20, 28]. This reduction was driven by the small gene pool of the migrating population, genetic drift during migration, and the founder effect. In contrast, African populations retained a larger effective population size, preserving higher levels of genetic diversity, including alleles that may be rare or absent in non-African populations [29]. As a result, the African genome harbours a far greater number of single nucleotide variants (SNVs) than non-African genomes [30]. This exceptional diversity provides unparalleled opportunities to reconstruct the origins of modern humans and identify disease-causing genetic variants [31]. For instance, apolipoprotein-1 (*APOL1*) gene variants are frequent in individuals of African origin, notably higher in individuals of West African descent, and non-existent in other non-African populations. Studies have demonstrated that African-American and West African individuals carrying *APOL1* high-risk variants have an increased risk of developing kidney diseases [32, 33]. These *APOL1* variants have also been associated with nephropathy in sickle cell disease (SCD)—a disease with the largest burden in sub-Saharan Africa [34]. A recent prostate cancer study in African men from the Men of African Descent and Carcinoma of the Prostate Network (MADCaP: with data from Ghana, Nigeria, Senegal, South Africa, and Uganda) identified novel association signals and haplotypes with large effect sizes that were not observed in non-African ancestries [35]. Thus, demonstrating the relevance of individuals with African ancestry in improving fine-mapping resolution [36].

## Advancing polygenic risk scores through African genomic representation

One of the advantages of conducting GWASs is the opportunity to develop polygenic risk scores (PRS) or polygenic scores (PGS), which can serve as markers for disease prediction and prevention in clinical care. PRS estimates an individual’s genetic risk for a phenotype by summing the number of risk alleles they carry, usually weighted by their effect sizes. It is well-developed and suited to predict breast and prostate cancer and type 2 diabetes in white European individuals, and allows the incorporation of non-genetic factors such as family history, lifestyle, and clinical examination outcomes for the prediction [37]. However, European-derived PRS could not predict prostate cancer in African men [38]. The predictive accuracy and utility of PRS for a population depends largely on the representation of that population in the datasets used in developing the PRS, that is, the prediction diminishes when the genetic distance between populations increases [8, 39, 40]. For instance, the polygenic score developed using UK Biobank European GWAS of 17 anthropometric and blood panel traits has lower predictive power when applied to non-European populations (4.5-fold lower in Africans than Europeans) [8]. Whereas African-derived Polygenic scores (PGSs) had better predictability for lipid traits in independent sub-Saharan Africans compared to the European-derived PGSs, despite having fewer SNPs [41]. Hughes and colleagues constructed PGSs for creatinine-based estimated glomerular filtration rate (eGFR) and revealed that Africa-specific PGSs outperformed East Asian ancestry and European ancestry scores for prediction into the African American test GWAS, despite >10-fold difference in sample size. Meanwhile, Africa-specific PGSs explained a low proportion of eGFR variance in West Africans (Ghana and Nigeria), similar to the East Asian and European ancestries [40]. This may reflect the differences in the environmental exposures and lifestyle, LD patterns, and genetic admixture in African Americans compared to continental Africa.

The existing African genomic and molecular datasets represent just the tip of the iceberg, offering only a fraction of the potential insights from the African populations (Fig. 1).The underrepresentation of individuals of African ancestry in GWAS limits the applicability of PRS across diverse populations, as most models are primarily based on European ancestry data [16, 42, 43]. Prioritising genomic diversity through increased representation of African genomes will help reduce the bias toward European populations in current GWAS datasets. Consequently, PRS could become more predictive and generalisable across different ancestries, addressing health disparities and fostering equitable precision medicine.

## Addressing genomic and demographic complexities in African populations

The H3Africa (Human Heredity and Health in Africa) consortium and many other studies have shown the importance of characterising African genetic diversity to enhance global health outcomes, uncovering insights into human ancestry, migration patterns, skin pigmentation, and strong natural selection, particularly in genes related to viral immunity, DNA repair, and metabolism along with complex patterns of ancestral admixture [4, 21, 22, 29, 44–46]. For example, while investigating the genetic diversity of Ugandan population groups, we uncovered a population substructure that is influenced by Eurasian and East African Nilo-Saharan gene flow and aligns with historical geographic origins despite significant migration and admixture. Formal admixture tests provided evidence of Eurasian gene flow into Uganda (back-to-Africa migration), corroborated by Eurasian mitochondrial and Y-chromosome haplotypes, as well as detection of Neanderthal ancestry, confirming recent gene flow from Eurasian populations [22].

The variability between and within African populations, characterised by the heterogeneity of effect sizes and differences in allele frequency and linkage disequilibrium (LD) patterns, poses unique challenges for genomic analysis [42]. Janivara and colleagues showed marked allelic heterogeneity in the genetic architecture of prostate cancer across sub-Saharan Africa driven by differences in allele frequencies and effect sizes influenced by recent mutations, genetic drift and population bottlenecks [4, 35, 41, 44, 47]. Addressing these complexities requires robust statistical approaches that account for population structure and admixture to mitigate false-positive association signals.

Environmental and lifestyle differences across African communities and countries further influence the effect sizes of genetic variants. In the UGR cohort, we estimated the narrow-sense heritability of 34 cardiometabolic traits, with LDL-cholesterol exhibiting higher heritability in Ugandans than in Europeans (54% versus 20%–43%), while height displayed lower heritability in Ugandans (49% versus 70%–80% in Europeans), reflecting differing genetic influences and environmental interactions. This cohort and other African-based genomic studies contribute invaluable data providing insight into the genetic architecture of diverse African populations [22]. It is well-known that heritability estimates of phenotypes differ across ancestries, impacting PRS performance and transferability [8, 25] (Fig. 1). For example, African American-derived PRS performed better in the South African Zulu cohort compared to multi-ancestry and European-derived PRS for LDL-cholesterol, HDL-cholesterol, and total cholesterol. Conversely, multi-ancestry PRS showed better performance for triglycerides, indicating the phenotype-dependent nature of PRS transferability [48]. Interestingly, the superior performance of African American-derived PRS observed in the South African Zulu cohort was not replicated in the Ugandan cohort, except for total cholesterol. Furthermore, the predictive power of PRS was generally lower in the Ugandan cohort compared to the South African Zulu cohort. Differences in allele frequency, age, and body mass index between the two cohorts as well as the urban residency of the South African Zulu cohort, likely contributed to this disparity [48]. In our recent meta-analysis of LDL-cholesterol in African individuals, we tested a hypothesis that environmental variables could drive het-erogeneity of effect sizes. Therefore, using environment-adjusted Meta-Regression of Multi-Ethnic Genetic Association (env-MR-MEGA), we demonstrated that including the proportion of rural versus urban status as a study-level environmental covariate accounted for effect size heterogeneity in diverse African populations [49]. Unlike European populations, where LD patterns are more homogeneous, the high genetic diversity within African populations necessitates accounting for local and regional genetic differences and environmental influences when developing PRS. Failure to do so will result in poorly predictive scores, limiting the clinical utility of PRS for African individuals. Moreover, genetic studies in Africa must carefully consider study design and sampling strategies that ensure well-powered, balanced case–control representation and diverse sampling across regions.

## Expanding phenotype data collection in Africa

There is a noticeable gap in the genomics of non-communicable diseases (NCDs) such as psychiatric, neurological and neurodegenerative disorders, sickle cell disease, cancers, and respiratory diseases in Africa. Genetic and phenotypic data for these and other conditions remain scarce. This gap limits the discovery of disease-causing variants, reduces the portability of non-African findings to African populations, biases PRS utility, and misses opportunities to uncover evolutionary pressures and protective mechanisms for diseases.

Despite the rising challenge of mental health disorders in Africa with a prevalence of 13% [50], genetic studies of these conditions remain underrepresented in the continent. This lack of representation may hinder innovation in diagnostics and drug development. NeuroGAP (Neuropsychiatric Genetics of African Populations-Psychosis) is one cohort addressing some of these challenges through the enrolment of 34, 000 participants from four African countries (Ethiopia, Kenya, South Africa, and Uganda) for a case–control GWAS of Schizophrenia and bipolar disorder [51].

**Figure 2:**
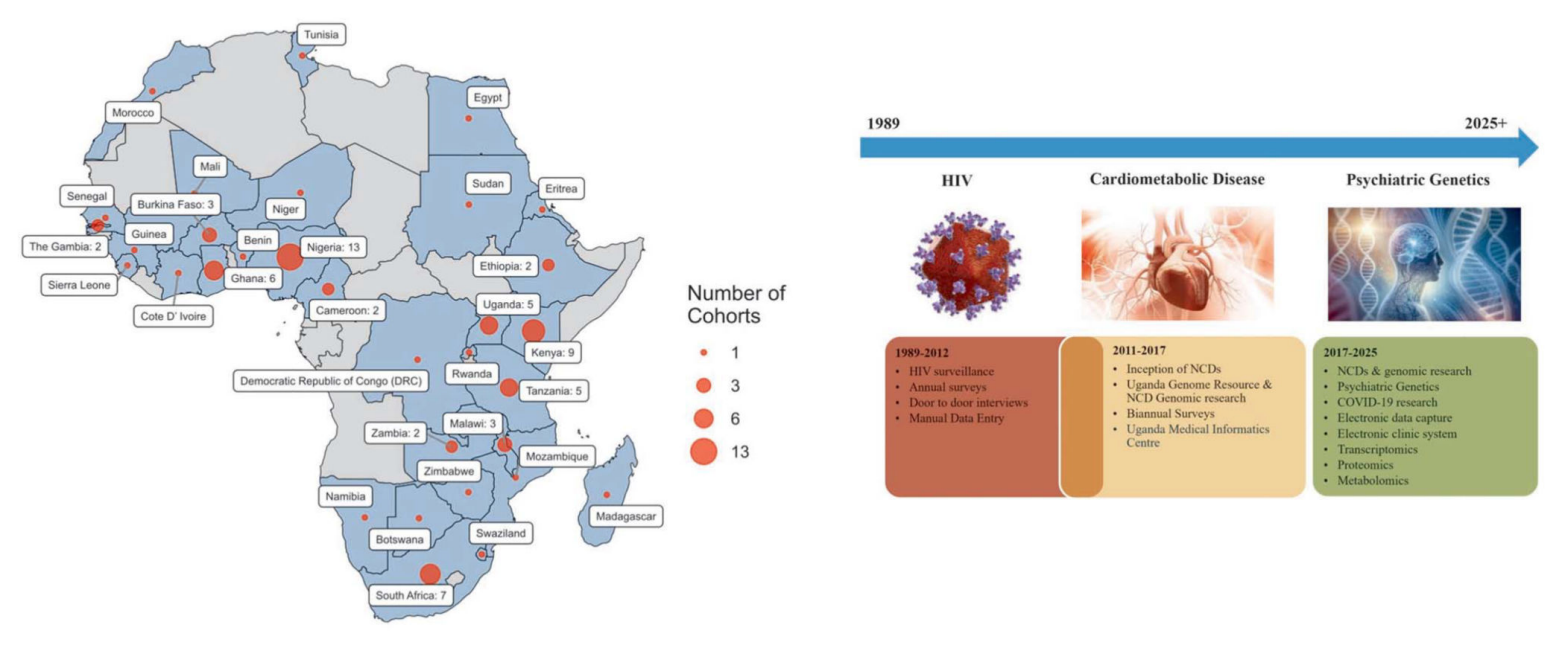
African countries with biobanks and/or cohorts with genetic datasets and the progress of Uganda genome resource. (A) African countries with biobanks and/or cohorts with genetic datasets. (B) Progress of Uganda genome resource since 1989.

In addition, the UGR which stemmed from the General Population cohort in 1989, to investigate the prevalence and incidence of HIV infection, incorporated genetics of cardiovascular diseases in 2015 [22]. Currently, UGR has evolved to include multi-omics datasets and psychiatric genetics (Fig. 2) [52]. Furthermore, the Stroke Investigative Research and Education Network (SIREN) is offering new perspectives on stroke prevention and treatment in Africa through its multicentre study sites in Ghana and Nigeria. They conducted the largest epidemiological study on stroke among continental Africans [53, 54] and recently identified novel genomic regions near *AADACL2* and *MIR4458HG* with significant protective associations against ischaemic stroke [55]. SIREN is one of the six cardiovascular working groups (known as CHAIR: Cardiovascular H3Africa Innovation Resource) within the H3Africa consortium [11].

Sub-Saharan Africa bears the largest burden of SCD globally, accounting for 270 000 out of 300 000 annual newborns worldwide [56]. Despite this burden in Africa, only two genome-wide association studies have been conducted on SCD in Africa [57, 58].

The various genomic projects, including the African Centre of Excellence for Genomics of Infectious Diseases (ACEGID) in Nigeria, UGR, CHAIR, Nigeria 100 K Genome Project, NeuroGAP, the Sickle Cell Disease Genomics of Africa (SickleGenAfrica) Network [1, 11, 51, 59–61], and many other studies, are poised to yield profound insights into the genetic determinants of many diseases in African populations (Fig. 2). These initiatives hold the potential for novel and functional genetic discoveries, that could drive the development of targeted interventions and treatments. Furthermore, continued investment in large-scale biobanks and integration of African genomic data into the broader global research landscape, will play a crucial role in improving the accuracy of disease risk predictions, improving drug response, and facilitating creation of personalised healthcare solutions.

Given the prevalence of many diseases in Africa, investigating disease associations in populations with the highest burden provides a unique opportunity to identify risk alleles that may be absent in other populations. This underscores the need for expanded genetic research on a broader range of phenotypes across the continent.

Studies in Africa often collect phenotypes with limited scope during participants’ enrolment and consent, which restricts opportunities for follow-up research and complicates efforts to harmonise phenotypes across studies or cohorts. Implementing H3Africa standardised case report forms for phenotype data collection could bridge this gap by improving data quality, consistency, and reliability [62]. More importantly, the availability of electronic health records will accelerate genomic studies in Africa to better understand the genetic contributions to health and disease. Electronic health records (EHRs) provide a rich source of data, including demographics, clinical diagnoses, disease progression, laboratory measurements, and treatment outcomes to improve healthcare quality [63]. They have played a crucial role in large genomic studies in high-income countries. Adoption of EHRs remains limited in many African countries due to reliance on paper-based systems and fragmented healthcare systems that lack unified national frameworks. Key challenges include inadequate infrastructure for collecting and managing EHRs, a shortage of skilled personnel in health informatics and bioinformatics, limited financial resources and a lack of prioritisation by policymakers [64, 65]. To address these challenges, it is essential to recognise the impact of EHRs on healthcare delivery, including their role in advancing precision medicine. Adoption policies must be accompanied by adequate funding and capacity building for all aspects of EHR data management. Achieving this will require a long-term commitment from the governments, health institutions, and their leadership, to ensure proper implementation and sustainability of EHR [66].

## Challenges and recent developments in African genomic research

The COVID-19 pandemic initially disrupted many ongoing genomic studies unrelated to COVID-19 as resources were redirected to prioritise severe acute respiratory syndrome coronavirus 2 (SARS-CoV-2) surveillance in Africa, and genomic laboratories repurposed for diagnostic testing. The urgent need for genomic surveillance of SARS-CoV-2 exposed the inadequate genomic infrastructure across the continent, prompting the African governments and stakeholders to acknowledge the importance of integrating genomics into disease surveillance [67–69]. This led to the establishment of genomic hubs and investments in sequencing platforms. The pandemic also fostered collaboration between African countries/scientists for data sharing and SARS-CoV-2 tracking [70]. Moreover, African public health and academic institutions strengthened their capacity to generate and analyse genomic data during this period, laying the groundwork for broader applications in genomic research.

Remarkable progress has been made in African Genomics through H3Africa and many other initiatives. H3Africa was a landmark initiative, funded by the National Institute of Health (NIH), USA and Welcome Trust in the UK, facilitated African-led genomic research to understand the genetic and environmental factors influencing diseases on the continent, established high-quality biorepositories, and built genomic research infrastructure and bio-informatic skills [10, 71]. This consortium studied ~118, 000 participants in 51 projects across 30 African countries between 2012 and 2022 (http://h3africa.org) [71]. In collaboration with Illumina, the consortium designed a ~ 2.3 M SNP H3Africa genotyping chip with a panel of genetic variants and clinically relevant variants selected from African sequence data. This chip has allowed genotyping for GWASs involving African individuals [41, 55, 58]. As African genomic datasets and research in the continent continue to expand, it is crucial to address barriers to scientific progress in Africa such as challenges in participant enrolment linked to historical mistrust due to past misuse and exploitation of their genomic data. By adopting meaningful community engagements that prioritise participant-centred approaches, trust can be rebuilt to improve participation in genomic research [31, 72]. The success of this approach is evident in the initiatives such as the H3Africa consortium and the UGR [60].

Many African countries have limited access to high-throughput genomics technologies, bioinformatics expertise and infrastructure, and funding necessary to conduct genomic studies and perform big data analytics. This lack of resources hampers efforts to generate robust datasets that capture the genetic diversity across Africa’s many ethnolinguistic groups. Collaboration between African and international researchers is essential to overcoming the current limitations.

Data Science for Health Discovery and Innovation in Africa (DSI-Africa) was established to leverage genomic data science for the development of innovative solutions that will transform health in Africa (https://dsi-africa.org/). NIH is funding this initiative and will build upon the existing H3Africa infrastructure to revolutionise data science in Africa. One key area being emphasised by DSI-Africa is building the capacity of African Scientists and fostering networks of African investigators. The initiative includes a data science platform and coordinating centre, four projects on the ethical, legal and social implications of data science research, seven data science research training programs and seven research hubs (https://dsi-africa.org/projects).

On October 1, 2024, Wellcome and the Chan Zuckerberg Initiative announced the establishment of the African Bioinformatics Institute (ABI) to build bio-informatic infrastructure and provide training to African scientists in order to advance genomics in Africa over a five-year period (https://wellcome.org/news/were-establishing-new-institute-advance-genomics-africa).

Genomics of kidney disease in Africa (KidneyGen Africa) is a new Pan-African partnership project awarded by the UKRI/Medical Research Council, poised to transform chronic disease research in Africa (https://www.kidneygenafrica.org). The initiative will facilitate large-scale chronic kidney disease genomic studies in collaboration with global consortia such as CKDGen and COGENT-Kidney and empower at least 100 early-career African scientists between 2024 and 2029.

Another initiative, the African Network of Genomic Centers of Excellence (GenCoE) in partnership with Roche, aims to expand Africa’s capacity in genomics. This initiative seeks to drive genomic discoveries for drug development, address global health inequities, and position African scientists as key contributors to shaping the future of global biomedical research (https://www.nature.com/articles/d44148-023-00052-z).

National governments across Africa must demonstrate a stronger commitment to genomics research to ensure the sustainability of many ongoing internationally funded studies and treatments in Africa. However, it appears that the lessons and insights gained during the COVID-19 pandemic about how a lack of investment in genomics research could affect human health have already been forgotten.

Addressing data ownership and equitable data sharing to benefit the communities and African researchers is another critical aspect of enhancing African genomic research [72]. This will require establishing governance structures, strengthening legal frameworks (including the African national and regional governments), respecting ethical considerations, and promoting data sharing. African communities and study participants must be part of the decision-making regarding the use of their data and policies and protocols must be in place to ensure that data handling, storage, sharing and reuse are tailored to the African context and ensure that the outcome of genomic research will benefit the Africans. Initiatives such as DSI-Africa and ABI will foster collaborations/partnerships that bring together African institutions, and researchers to strengthen local expertise and infrastructure. Such collaborations will ensure the sharing of resources and expertise while empowering African scientists to lead their research programmes.

The expansion of genomic research to encompass African populations, especially those ethnolinguistic groups currently underrepresented in research initiatives, is indispensable in our global pursuit of precision medicine.

## Conclusions

Expanding genomic research in African populations is paramount due to the continent’s remarkable vast genetic diversity, which remains significantly underrepresented in global studies. The inclusion of African genomic data in global resources is far-reaching, as they have the potential to drive precision medicine. Different initiatives and consortia have played pivotal roles in unveiling profound insights into African genetic diversity and disease susceptibility. It is essential to continue to foster collaborative efforts between African and global research communities to close the gap of the historical lack of representation in genomic databases and ensure that advances in genomics are inclusive and equitable.
